# ChronoimmunoTOX: A Single-Institution Retrospective Study on How the Time of Administration Impacts Immune Checkpoint Inhibitor Efficacy and Toxicity in Melanoma

**DOI:** 10.3390/jcm15010069

**Published:** 2025-12-22

**Authors:** Alessandro Nepote, Gilles Burghgraeve, Martino Pedrani, Anderson Junior Gomez Ramos, Isabella Saporita, Vito Spataro, Vittoria Espeli, Ricardo Pereira Mestre, Dario Sangiolo, Martina Imbimbo, Cristina Mangas

**Affiliations:** 1 Istituto Oncologico Svizzera Italiana, Ente Ospedaliero Cantonale, Ospedale Regionale Bellinzona e Valli, 6500 Bellinzona, Switzerland; alessandro.nepote@unito.it (A.N.); gilles.burghgraeve@usi.ch (G.B.); martino.pedrani@eoc.ch (M.P.); junioranderson.gomezramos@eoc.ch (A.J.G.R.); isabella.saporita@unito.it (I.S.); vito.spataro@hin.ch (V.S.); vittoria.espeli@eoc.ch (V.E.); ricardo.pereiramestre@eoc.ch (R.P.M.); martina.imbimbo@eoc.ch (M.I.); 2 Dipartimento di Oncologia, Università di Torino, Regione Gonzole 10, 10043 Orbassano, Italy; dario.sangiolo@unito.it; 3 Candiolo Cancer Institute FPO-IRCCS, Strada Provinciale 142 km 3.95, 10060 Candiolo, Italy; 4 Dermatology Department Ente Ospedaliero Cantonale, Ospedale Regionale Bellinzona e Valli, 6500 Bellinzona, Switzerland

**Keywords:** Chronoimmunotherapy, melanoma, Anti PD-1, anti CTLA-4, immune adverse events

## Abstract

**Background**: The timing of immune checkpoint inhibitor (ICI) administration may influence clinical outcomes, incidence, and severity of immune-related adverse events (irAEs), but evidence remains limited. **Methods**: We conducted a retrospective analysis of 41 patients with advanced melanoma treated with combined ipilimumab and nivolumab at the Istituto Oncologico della Svizzera Italiana between 2018 and 2024. Infusions completed before 2:00 p.m. were classified as morning (AM). Patients receiving ≥50% of doses in the morning were assigned to the AM group; the remaining patients comprised the afternoon (PM) group. **Results**: Twenty-one patients were included in the AM group and twenty in the PM group. Median progression-free survival (PFS) was not reached in the AM group, compared with 7.8 months in the PM group (univariate HR 0.29, 95% CI 0.12–0.70; *p* = 0.006). Overall survival (OS) was also significantly improved in the AM group (univariate HR 0.25, 95% CI 0.08–0.80; *p* = 0.019). The overall incidence of irAEs was similar between groups. However, systemic immunosuppression for grade ≥ 2 toxicities was more frequently required in the PM group (80% vs. 52%, *p* = 0.06). **Conclusions**: In this retrospective cohort, morning administration of ICIs was associated with improved PFS and OS in patients with advanced melanoma. While irAE incidence was comparable between groups, patients treated in the afternoon more often required systemic immunosuppression.

## 1. Introduction

The introduction of immune checkpoint inhibitors (ICIs) has deeply changed the management and prognosis of patients with metastatic melanoma (MM) over the past decade, unleashing T cells’ ability to mediate antitumor responses by targeting cytotoxic T-lymphocyte-associated protein 4 (CTLA-4) and programmed death-1 (PD-1) signals [1,2].

However, about 50% of treated patients do not achieve sustained benefits from immunotherapy and will therefore progress, highlighting the need for a deeper understanding of the biological mechanisms that drive primary and secondary resistance to immunotherapy [3,4].

The efficacy and toxicity of immunotherapy in melanoma is linked to complex interactions between host-related, disease-related, and external factors. Despite several efforts, we still lack strong predictive biomarkers that could assist in patients’ selection [5]. The interferon-gamma (IFN-γ) signature has been identified as potentially prognostic and predictive in stage III melanoma, but its use is not standard in clinical practice [6]. Several groups are leveraging on machine learning (ML) tools to build predictive algorithms: Yoo and colleagues published their experience with SCORPIO, a ML system that utilizes routine blood tests to predict outcomes of ICI therapy across various cancer types, including melanoma [7]. Despite these findings, there is still need for widely applicable biomarkers to predict treatment outcomes.

Toxicities derived from ICIs are non-negligible [2,8]. Incidence of irAEs varies significantly depending on the treatment regimen. While combination immunotherapy seems to offer substantial improvements in overall survival (OS) and progression-free survival (PFS), it also bears an increased risk of significant toxicities, with severe (G3-4) irAEs affecting about 60% of patients compared to 20% of patients treated with anti-PD-1 monotherapy [3,4]. Toxicities and their management affect patient quality-of-life treatment adherence and represent a significant financial burden for healthcare systems [9,10]. To date, no biomarkers are available to predict the likelihood of developing irAEs, nor are there established strategies to reduce their incidence. In this framework, gene expression profiling of peripheral blood cells showed promising results in predicting treatment outcomes during anti-PD-1 therapy; however, the study did not consider the immune system composition at the time of blood withdrawal [11,12].

Immunochronobiology is the study of how biological rhythms influence immune responses, and it may provide novel insights into optimizing treatment timing to enhance efficacy and minimize toxicity.

Circadian rhythm (CR) plays a key role in regulating multiple physiological functions, including immune cell activity, by influencing processes such as T-cell activation and cytokine secretion, which are critical for immunotherapy effectiveness. Disrupted clock gene expression, commonly observed in melanoma tumors, has been shown to impair tumor immune surveillance and reduce the efficacy of ICIs [13]. As such, immunochronotherapy, which consists of the alignment of treatment administration with circadian rhythms, may be an effective strategy to reduce irAEs while enhancing overall treatment outcomes.

The first administration of ICI treatment seems crucial for evaluating the impact of administration timing on outcomes. During this period, the highest drug concentrations are achieved, leading to peak immune activation, including robust T-cell activation and cytokine responses, thereby optimizing the potential for therapeutic benefits [14,15,16].

The first treatment cycles also correspond to the highest incidence of irAEs, as observed in clinical trials [17] and confirmed in comparative analyses as well as real-world data sets [18,19,20]. This is especially true for the anti-CTLA-4/anti-PD-1 combination, while pooled analysis revealed that the onset of severe irAEs may be delayed compared to all irAEs, especially with anti PD/L-1 monotherapy [21].

Preclinical and retrospective analyses have revealed that timing of ICI administration could probably influence clinical outcomes, including overall response rate (ORR), progression-free survival (PFS), and overall survival (OS); however, no correlation with toxicity has been described [14,22,23,24].

In the recent nine-year update of the Checkmate 238 trial, the authors reported, in a post hoc analysis, a higher incidence of irAEs leading to discontinuation in the group treated with ipilimumab after 1 p.m. compared with the group treated in the a.m. (37.7% vs. 26.6%), only when ≥75% of the doses of either nivolumab or ipilimumab were administered before or after 1:00. However, in-depth statistical analysis was not provided [25].

Based on these premises, we conducted a monocentric, retrospective study, of which the aim is to evaluate the impact of the timing of ICI administration on outcomes and irAEs. The study included patients with advanced cutaneous MM treated at the Istituto Oncologico della Svizzera Italiana (IOSI) between 2018 and 2024.

## 2. Material and Methods

This monocentric, retrospective study evaluated all patients with stage III or stage IV non-uveal melanoma treated with nivolumab and ipilimumab between January 2018 and September 2024 at IOSI. Data—including sociodemographic, clinical-pathological, and treatment details as well as toxicity profiles—were extracted from both electronic and paper records. The study adhered to ethical standards (Project ID 2024-02005, CE 4699) and the Declaration of Helsinki. We included patients with confirmed histological diagnosis of cutaneous, mucosal, acral, or unknown primary melanoma, while we excluded uveal melanoma. We included 3 cases of not-otherwise-specified (NOS) melanoma, for which we could exclude the uveal origin based on molecular profiling.

A more detailed description of the inclusion criteria, exclusion criteria, and variable definitions can be found in Supplementary Method S1.

Patients were classified into AM (morning) or PM (afternoon) groups based solely on the timing of the first four doses of ipilimumab and nivolumab, excluding the time of administration of nivolumab maintenance therapy doses.

We categorized an infusion as “morning” if completely administered before 2:00 PM, considering a 1 h delay from prescription to infusion. Patients who received at least two morning infusions (at least 50% of their infusions occurred in the morning) during their first four doses of ipilimumab–nivolumab were classified into the AM group. Conversely, patients with fewer than 50% of the doses in the morning were categorized into the “afternoon” (PM) group. Patients who received fewer than four infusions due to toxicity or progression were classified in the AM group if at least 50% of the doses received were in the “morning”. Patients who received a single infusion were classified according to that single infusion.

The primary endpoint was PFS, while secondary endpoints included OS, best response at first radiological evaluation according to RECIST if available, and the severity of irAEs, according to CTCAE. v.5. All initial radiological evaluations were performed using FDG PET/CT with iodine contrast and reviewed by a multidisciplinary board. A detailed definition of the outcomes can be found in Supplementary Method S2.

Descriptive statistics and inferential tests were employed. Pearson’s chi-square test compared categorical variables, and the Kruskal–Wallis test was used for continuous measures. PFS and OS were estimated by the Kaplan–Meier method and differences between groups were examined with the log-rank test. Univariate Cox proportional hazards models assessed the impact of “time of ICI administration” along with clinical and demographic variables such as age, disease extension, sex, BRAF mutation status, and brain metastases. Variables identified as significant (*p* < 0.05) or deemed clinically important a priori (e.g., BRAF status and number of metastatic sites) were included in multivariate Cox regression analyses. Sensitivity and subgroup analyses were also carried out to test the robustness of the findings. Given the prognostic value of BRAF mutation, we conducted a prespecified sensitivity analysis restricted to BRAF-mutated patients to assess whether the effect of ICI administration timing persisted within this subgroup. Post hoc analysis excluding patients carrying brain metastasis and patients with more than three metastatic sites were also performed. A detailed statistical analysis can be found in Supplementary Method S3.

The results are presented as hazard ratios (HR) with 95% confidence intervals (CI95%). All statistical analyses were carried out using R statistical software version 4.4.1 and Jamovi statistical software version 2.5.6.

## 3. Results

We collected data from 41 patients with stage III or stage IV melanoma treated at IOSI with the combination of nivolumab and ipilimumab from 2018 to 2024. Based on the criteria outlined in the methods section, 21 patients were assigned to the AM group and 20 to the PM group.

Table 1 summarizes the demographic and disease characteristics of the study population. The majority of patients were male (*n* = 23; 56%), had an Eastern cooperative oncology group (ECOG) performance status of 0 (*n* = 37; 90%), and exhibited normal LDH levels at baseline (*n* = 33; 80%). Both groups were balanced for major prognostic factors, including the presence of brain metastases and the number of metastatic sites, except for BRAF mutation status, which showed a statistically significant difference (PM 80% vs. AM 40%, *p* = 0.02), with a higher prevalence in the PM group. More than half of patients received ipilimumab and nivolumab as a first-line treatment (32 of 41; 76%).

Three patients (7% of total) were classified as potentially resectable stage III but declined upfront surgery; therefore, they were treated with the neoadjuvant dose of ipilimumab, 1 mg/kg, and nivolumab, 3 mg/kg, while the rest of the cohort received the standard doses of ipilimumab, 3 mg/kg, and nivolumab, 1 mg/kg.

A small percentage of patients had received previous treatment: six patients (15%) were treated initially with BRAF/MEK-targeted therapy, while three patients (7%) received single-agent ICI (pembrolizumab or nivolumab) therapy; therefore, they were treated with ipilimumab and nivolumab as a second-line treatment (Table 1).

The median follow-up duration was 29.7 months (range: 3.1–60.4). At the data cut-off, the disease control rate (DCR) was significantly higher in the AM group (93%) compared to the PM group (57%, *p* = 0.01), with nine patients in the AM group achieving complete response (CR) versus five in the PM group (Table 1).

**Figure 1:**
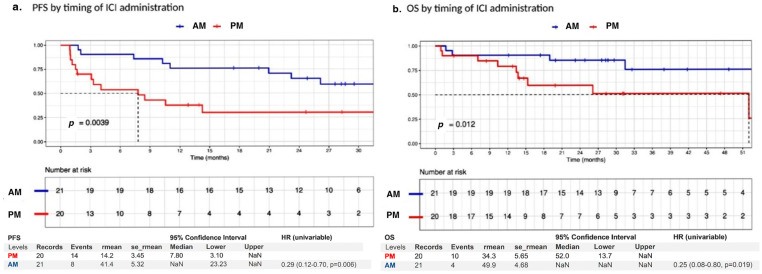
PFS (**a**) and OS (**b**) in AM vs. PM patients.

The primary endpoint of the study was achieved, with the AM group demonstrating a significant reduction in the risk of progression and death compared to the PM group (UV PFS HR 0.29, 95% CI: 0.12–0.70, *p* = 0.006; Figure 1). The median PFS was not reached in the AM group (95% CI: 23.2–NR) compared to 7.8 months (95% CI: 3.10–NR) in the PM group, as shown in Figure 1.

The PFS benefit for the AM group remained significant in the multivariate analysis (Table 2) and was further supported by sensitivity analysis (Table S1). Similarly, the risk of death was significantly reduced in the AM group compared to the PM group (UV OS HR 0.25, 95% CI: 0.08–0.80, *p* = 0.019; Figure 1), and the OS benefit for the AM group was maintained in the multivariate analysis (Table 2). In both analyses, the interaction test showed no significant impact of BRAF status or the number of metastatic sites on the effect of administration timing (Table 2). In a prespecified analysis restricted to BRAF-mutated patients, morning ICI administration remained associated with longer PFS (HR 0.20; *p* = 0.047) (Table S2). Post hoc analysis excluding patients with SNC metastasis or with more than three metastatic sites yielded similar results (Table S3).

**Figure 2:**
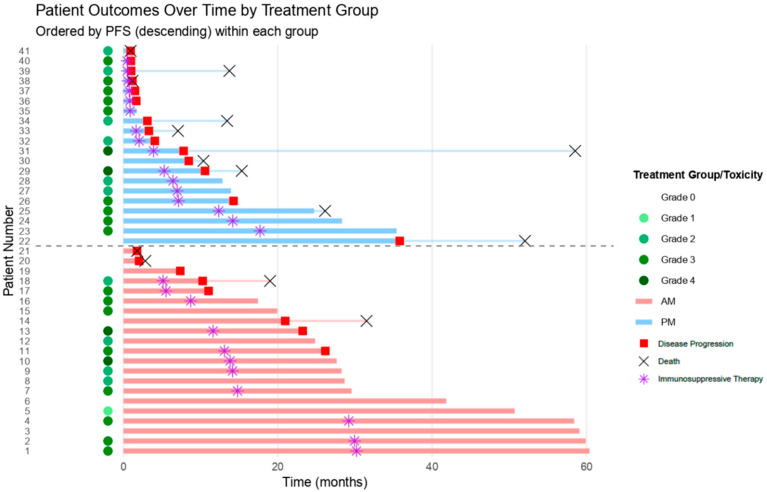
Swimmer plot reporting irAEs and respective treatment, according to time of administration and ordered by PFS.

The overall incidence of irAEs was similar between groups. However, grade ≥ 2 irAEs requiring immunosuppressive treatment, including steroids or biological drugs (anti IL-6) as a first-line treatment for toxicities, were more common in the PM group (80% vs. 52%, *p* = 0.06), potentially indicating greater irAE severity or more complex management in this group (Table 1). The majority of patients were treated with corticosteroids as a first-line treatment (AM: 11.52%, PM: 15.75%, *p* = 0.04). A summary of treatment outcomes and irAEs for all patients is provided in Figure 2 and Table 1.

Exploratory analyses were conducted stratifying patients by clinical and demographic characteristics to test the effect of ICI administration timing across different patient subgroups. In the multivariate Cox regression analysis for PFS, a significant interaction was identified between patient age and timing of ICI administration (*p* = 0.035), suggesting that age may modulate the effect of treatment timing (Table S4).

As a result, pairwise subgroup comparisons were performed using the Holm adjustment method. A statistically significant difference was observed only between patients aged < 60 years treated in the morning (AM) versus those treated in the afternoon (PM) (*p* = 0.003), with PM-treated patients exhibiting a 12-fold-higher risk of progression or death compared to their AM counterparts (HR 12.05, 95% CI: 2.35–61.65; Table S5a,b).

Univariable Cox regression analyses for PFS stratified by age further confirmed this finding: among patients aged < 60 years, AM administration was associated with a markedly reduced risk of progression or death (median PFS: AM group not reached vs. PM group 9 months; *p* = 0.006). In contrast, no significant difference in PFS was observed in patients aged ≥ 60 years (HR 0.89, 95% CI: 0.25–3.17; *p* = 0.854) (Table S5c,d, Figure S1).

Patients under 60 were more likely to carry a BRAF mutation (70%), while older patients had a higher burden of comorbidities and were more frequently affected by brain metastases. Clinical and demographic characteristics stratified by age are reported in Table S6.

## 4. Discussion

Our single-center retrospective study suggests that patients with advanced or metastatic melanoma derived greater benefit from receiving immunotherapy in the morning compared to the afternoon, with statistically significant differences in PFS and OS. We also noted a numerical difference in toxicity severity: patients who received immunotherapy in the morning required less systemic immunosuppressive treatment for grade ≥ 2 irAEs, including steroids.

Our findings on efficacy align with the understanding that the immune system’s composition in the blood and lymph nodes varies throughout the day according to circadian rhythms [26]. In a preclinical study by Wang et al. [26], mice with melanoma tumors were treated with ICIs, and T-cell activation and cytokine secretion were found to follow a circadian pattern peaking in the “active phase” of the mice, which corresponds to our evening. Additionally, tumors grew less when treated in the evening (ZT13) compared to the morning (ZT1), demonstrating that the efficacy of treatment aligns with circadian rhythms. The administration of anti-PD-1 therapy at ZT13 enhanced CD8+ T-cell activation and degranulation, leading to increased production of IFNγ and granzyme B compared to administration at ZT1 [26]. These preclinical observations are supported by other retrospective studies in humans, which have also reported a survival benefit for melanoma patients treated with immunotherapy during the morning phase.

In the MEMOIR trial, patients treated after 4:30 PM exhibited worse OS (49% vs. 68%) and a reduced ORR compared to those treated earlier [22]. Consistently, in the study conducted by Yeung and colleagues, patients who received immunotherapy in the morning (before 12:00 PM) had improved PFS and OS [23]. Moreover, Karaboué et al. summarized all retrospective studies conducted to date, confirming this trend [14]. More recently, a large bi-continental retrospective study in patients with advanced non-small-cell lung cancer also confirmed this trend, showing significantly longer survival among those receiving immuno-chemotherapy before 11:30 AM compared with later administration [27]. Despite this overall positive trend, a recent systematic review highlighted that the evidence remains heterogeneous and sometimes discordant, with variations in patient populations, cancer types, ICI regimens, and cut-off times for early versus late administration [28].

However, none of these studies identified differences in toxicity [14,22,29]. Indeed, we observed a trend toward higher toxicity severity in the PM group, as evidenced by the increased need for secondary immunosuppressive agents to manage grade ≥ 2 adverse events. These findings indicate potentially greater morbidity from immunotherapy in patients treated in the afternoon. The recent study by Ascierto et al. yielded findings consistent with ours, but the reported benefit in PFS was limited only to the anti-CTLA-4 group, whether considering the first dose, the first two doses, or >75% of scheduled doses [25]. In our small cohort, the predominance of advanced-stage disease and the use of ipilimumab plus nivolumab could highlight the potential relevance of administering initial immunotherapy doses in the morning.

Data on efficacy in our cohort might be biased by the use of immunosuppressive treatments for management of irAEs, which was more frequent in the afternoon cohort (Table 1).

The correlation between treatment response and toxicity remains an area of ongoing investigation [17]. Certain events could be predictive of efficacy when a response against an antigen shared between healthy tissue and melanoma can be identified (i.e., skin toxicities). However, the onset of other severe irAEs, such as hepatitis or pneumonia, does not always correlate with treatment efficacy. [5,30].

Higher peak corticosteroid doses are associated with worse outcomes [31], while the cumulative dose does not seem to significantly affect them. The impact of secondary immunosuppressive agents on antitumor activity remains uncertain. Although anti-IL-6R agents, such as tocilizumab, have been shown to preserve the antitumor effects of anti-PD-1 agents, some evidence suggests that steroid-sparing regimens may worsen patient prognosis, especially when anti TNF-alpha agents are adopted [30,31,32].

Our study may indicate that precise regulation of ICI administration timing could be an effective strategy to optimize both the efficacy and toxicity profiles of these therapies. Currently, published studies remain heterogeneous regarding the proportion of number of infusions and the accurate time cut-offs used to define “morning” and “afternoon” administration schedules. From a biological perspective, it is also unclear whether specific T-cell subpopulations are underrepresented at certain time points, potentially influencing the pharmacokinetics of anti-PD-1 and anti-CTLA-4 agents [14,22,29].

The threshold defining an infusion as “late” was pragmatically set between 12 PM [33], 12:54 PM [34], 1:00 PM [23,35], and 2:00 PM [29], extending up to 4:00–4:30 PM [36,37,38,39,40] across multiple cancer histologies, based on criteria originally derived from vaccine efficacy studies [41,42,43]. In the systematic review by Nagy et al. [28], cut-off times varied widely across studies, ranging from 11:30 AM to 4:30 PM. This lack of standardized definition for “morning” and “afternoon” administration compromises comparability between studies and reduces the robustness of the conclusions. In the meta-analysis by Landré et al. [44], 2:00 PM emerged as the most discriminative cut-off point, with infusions administered before this time being associated with superior efficacy compared to those given between 2:00 PM and 4:00 PM.

Additionally, the pharmacodynamic profile of these agents remains unclear and may clarify whether the absolute number of time-sensitive doses plays a critical role in treatment outcomes. According to previous studies, ICIs have an elimination half-life ranging from 14 to 27 days, with steady-state concentrations typically achieved after 2–4 months [15]. Based on this pharmacokinetic profile, the first four doses are likely sufficient to provide an initial immunological imprinting. Moreover, a median of 32.4 weeks is required to reduce T-cell PD-1 occupancy by 50% following nivolumab discontinuation, supporting the notion that the initial doses induce a long-lasting effect, with subsequent administrations maintaining this plateau [16]. CTLA-4 inhibitors, in contrast, have demonstrated a clear dose and exposure–response correlation, which is also associated with increased toxicity, whereas such a correlation has not been consistently confirmed for anti-PD-1 agents [45]. Importantly, pharmacokinetics alone does not fully explain potential differences in efficacy based on the time of day of administration. Preclinical and clinical data suggest that the timing of initial infusions may modulate circadian-driven immune activity within the tumor microenvironment, influencing treatment outcomes independently of plasma drug levels [46].

In our study, we integrated these observations by focusing on the first four doses of nivolumab and ipilimumab, selecting 2:00 PM as the cut-off to define “late” ICI administration. We considered two administrations as the minimum requirement to achieve biologically relevant steady-state exposure, from which we derived the 50% threshold applied in our analyses.

Although pharmacokinetic and pharmacodynamic data for ICIs suggest that the first one or two infusions may be sufficient, the lack of prospective and randomized evidence prevents definitive recommendations.

Finally, we hypothesize that additional factors could contribute to enhancing the effect of morning administration. To explore this, we analyzed different patient subgroups, including age: we reported an unexpected benefit for younger patients. While advanced age is a well-known negative prognostic factor, several reports have shown that older patients may still derive substantial greater benefit from immunotherapy [47]. It is therefore possible that in elderly patients, timing of administration is less critical than in younger individuals.

Our findings must be interpreted cautiously due to several important limitations. The retrospective design and small cohort (N = 41) introduce potential selection biases and limit generalizability, as patients receiving morning infusions may have had better performance status or more favorable disease characteristics not captured in our analysis. Unmeasured confounders including prior therapies, disease burden, and supportive care measures could have influenced both treatment scheduling and outcomes Additionally, the single-center design may introduce institutional biases not representative of other clinical settings.

To address some of these issues, we applied strict inclusion criteria to create a more homogeneous study population, conducted sensitivity analyses to mitigate potential biases, and compared our results with published literature on chronotherapy.

Specifically, we performed sensitivity analyses, excluding patients with an ECOG PS > 1 (*n* = 4) to reduce confounding from poor-prognosis cases, and we used multivariate Cox regression models to adjust for key prognostic factors such as BRAF mutation status and metastatic burden.

Although the PM group had a higher proportion of patients with BRAF mutations, we showed no significant impact of this variable efficacy. Moreover, in a prespecified analysis restricted to BRAF-mutated patients, morning ICI administration remained associated with longer PFS, indicating that imbalance in BRAF status is unlikely to account for the observed effect. Moreover, post hoc analysis excluding patients with SNC met or with more than three metastatic sites yielded similar results further supporting our findings.

Our study did not show any robust correlation between the “late” doses and the overall incidence of toxicities. Interestingly, although most adverse events were G2, patients receiving afternoon infusions more frequently required systemic immunosuppression drugs. This observation suggests that CTCAE clinical grading may not fully reflect the underlying immunological severity of an irAE. Moreover, distinct T-cell subsets are known to drive different immune-related toxicities [48] and certain populations, particularly Th17 CD4^+^ cells, have been associated with reduced corticosteroid sensitivity [49] potentially necessitating second-line immunosuppressive therapy [50,51].

These findings raise the possibility that circadian variation in the activation of specific T-cell compartments could influence both the spectrum and intensity of irAEs, as well as the need for targeted immunosuppression, although direct clinical evidence remains limited.

We also attempted to identify specific subpopulations who may benefit more from “morning” ICI treatment. In our analysis, younger patients seem to benefit more, although wide confidence intervals and limited statistical correction due to the small sample size strongly affect the interpretation of our results.

Despite the limitations, our study represents a first attempt to confirm the benefit of chronoimmunotherapy on efficacy in a highly selected population of metastatic or potentially resectable stage III patients with melanoma treated with ipilimumab and nivolumab, with an additional focus on toxicity.

Our approach adopted specific time windows, informed by clinical and preclinical evidence, indicating that morning administration may offer superior efficacy and safety compared to afternoon dosing; however, the implementation of such timing strategies can entail notable logistical challenges within clinical settings.

## 5. Conclusions

We present our six-year experience treating patients with metastatic melanoma with ipilimumab and nivolumab. Our findings suggest that patients receiving ICIs in the morning were less likely to experience disease progression and demonstrated significant improvements in both PFS and OS. Conversely, patients treated in the afternoon showed a higher likelihood of requiring steroids or other immunosuppressive agents to manage severe toxicities. Immune chronobiology represents a promising and emerging field in oncology, with the potential to optimize treatment outcomes for patients receiving ICIs, although it may pose logistical challenges. We encourage the international scientific community to promote prospective multicenter studies to endorse these findings and confirm if timing truly matters.

## Figures and Tables

**Table 1:** Clinical characteristics, treatment responses, and toxicity.

	Total	AM Group (*n* = 21, *n*, %)	PM Group (*n* = 20, *n*, %)	Pearson Test
**Sex**				*p* = 0.44
*F*	18 (44)	7 (33)	11 (55)	
*M*	23 (56)	14 (67)	9 (45)	
**ECOG PS**				*p* = 0.96
*0–1*	37 (90)	19 (90)	18 (90)	
*>1*	4 (10)	2 (10)	2 (10)	
**Age**				*p* = 0.87
<60	22 (54)	11 (52)	11 (55)	
≥*60 y*	19 (46)	10 (48)	9 (45)	
**Stage at diagnosis**				*p* = 0.32
*I (A,B)*	4 (10)	3 (14)	1 (5)	
*II (A,B,C)*	11 (26)	5 (24)	6 (30)	
*III (A,B,C,D)*	10 (24)	3 (14)	7 (35)	
*IV*	16 (40)	10 (48)	6 (30)	
**Stage at study entry**				*p* = 0.34
*III*	3 (7)	2 (10)	1 (5)	
*IV*	38 (93)	19 (90)	19 (95)	
*M1a*	3 (7)	0 (0)	3 (15)	
*M1b*	3 (7)	2 (10)	1 (5)	
*M1c*	13 (36)	9 (47)	4 (20)	
*M1d*	19 (50)	8 (42)	11 (60)	
**N° of metastatic sites at study entry**				*p*= 0.07
*<3*	28 (68)	17 (81)	11 (55)	
*>3*	13 (32)	4 (19)	9 (45)	
**BRAF V600 mutational status**				***p*= 0.02**
*Non-mutated*	18 (44)	13 (60)	5 (20)	
*Mutated*	23 (56)	8 (40)	15 (80)	
**Brain metastasis**				*p* = 0.28
*No*	22 (54)	13 (62)	9 (45)	
*Yes*	19 (46)	8 (38)	11 (55)	
**LDH**				*p* = 0.48
*<UNV*	8 (20)	5 (24)	3 (15)	
*>UNV*	33 (80)	16 (76)	17 (85)	
**Prior Treatment**				*p*= 0.97
*Anti BRAF–MEK inhibitors*	6 (15)	3 (14)	3(15)	
*Anti PD-1*	3 (7)	1 (5)	2 (10)	
*None*	32 (78)	17 (80)	15 (75)	
**Best response at first radiological evaluation**				
*CR*	14 (34)	9 (43)	5 (27)	*p* = 0.04
*PR*	13 (32)	8 (38)	5 (27)	
*SD*	1 (2)	1 (4)	0 (0)	
*PD*	9 (22)	1 (4)	8 (44)	
*NE*	4 (10)	2 (8)	2 (8)	
***DCR***	29 (70)	18 (93)	11 (57)	***p* = 0.01**
**Site of toxicities**				
*Liver*	11 (27)	5 (23,8)	6 (30)	*p* = 0.65
*Skin*	7 (17)	5 (23,8)	2 (10)	*p* = 0.24
*Gastro-intestinal*	6 (15)	2 (9,5)	4 (20)	*p* = 0.34
*Endocrine*	9 (22)	5 (23,8)	4 (20)	*p* = 0.77
**Toxicities grade, CTCAE 5.0**				
*G0–G1*	10(24.4%)	7 (33)	3 (15)	*p* = 0.17
*G2–G4*	31 (75.6%)	14 (67)	17 (85)	
*G0–G2*	20 (49.8%)	11 (52)	9 (65)	*p* = 0.64
*G3–G4*	21 (51.2%)	10 (48)	11 (55)	
First-line treatment for toxicities *	27 * (100)			*p* = 0.06
*Steroids*	26 (96)	11 (52)	15 (75)	***p* = 0.04**
*Tocilizumab*	1 (4)	0 (0)	1 (5)	
*None*	14	10 (48)	4 (20)	
**Second-line treatment for toxicities**	4 * (100)			
*Infliximab*	1 (25)	1 (50)	0 (0)	
*Tocilizumab*	3 (75)	1 (50)	2 (100)	
**Steroid dosage**	26 * (100)			
*1 mg/kg*	23 (88)	10 (83)	13 (93)	*p* = 0.31
*2 mg/kg*	3 (12)	2 (16)	1 (7)	
Steroid therapy duration *	26 * (100)			*p* = 0.08
*<3 months*	7 (27)	1 (9)	6 (40)	
*>3 months*	17 (65)	8 (72)	9 (60)	
*Not reported*	2 (8)	2 (19)	0 (0)	

* percentages are calculated considering this value as 100%. Abbreviations: CI = confidence interval; HR = hazard ratio; ECOG PS = Eastern cooperative oncology group performance status, n° = number, LDH = lactate dehydrogenase, UNV = upper normal value, CR = complete response, PR = partial response, SD = stable disease, PD = progression disease, NE = not evaluable, DCR disease control rate, CTCAE 5.0 = Common Terminology Criteria for Adverse Events 5.0.

**Table 2:** Univariate and multivariate Cox proportional hazard models.

		PFS		OS	
Prognostic Variable	Levels	Univariate Analysis	Multivariate Analysis	Univariate Analysis	Multivariate Analysis
Total N. 41		HR (95% CI), *p*-Value	HR (95% CI), *p*-Value	HR (95% CI), *p*-Value	HR (95% CI), *p*-Value
ECOG PS	0–1	-	-		
	>1	1.81 (0.53–6.14) *p* = 0.34		0.87 (0.11–6.77), *p* = 0.896	
AGE	<60	-	-		
	≥60	1.33 (0.57–3.10) *p* = 0.51		1.63 (0.54–4.94), *p* = 0.389	
SEX	F	-	-		
	M	0.56 (0.24–1.30) 0.18		0.73 (0.25–2.13), *p* = 0.568	
N° of metastatic sites	≤3	-	-		
	>3	2.55 (1.10–5.96) *p* = 0.030	1.78 (0.71–4.44) *p* = 0.2190	2.17 (0.72–6.54), *p* = 0.168	1.38 (0.41–4.59) *p* = 0.60
BRAF V600 mutational status	no	-	-		
	yes	1.75 (0.73 4.17) *p* = 0.21	1.04 (0.40–2.67) *p* = 0.935	1.35 (0.45–4.07), *p* = 0.590	0.59(0.16–2.10) *p* = 0.42
Brain metastasis	no				
	yes	1.58 (0.67–3.70) *p* = 0.28		3.29 (0.98–11.03), *p* = 0.053	
Time of administration	PM				
	AM	0.29 (0.12–0.70) *p* = 0.006 **	0.35 (0.14–0.93), *p* = 0.034 *	0.25 (0.08–0.80), *p* = 0.019 **	0.22 (0.05–0.88), *p* = 0.032 **
Test for interaction					
N° of metastatic sites	*p*-value			*p*-value	
	0.8845074			0.5091154	
BRAF mutation status	*p*-value			*p*-value	
	0.2025199			0.3014212	

Abbreviations: OS = overall survival (from treatment start to death); PFS = progression-free survival (from treatment start to first progression); CI = confidence interval; HR = hazard ratio; ECOG PS = Eastern cooperative oncology group performance status. Prognostic variables were included in the multivariable model if *p*-value ≤ 0.05 *. BRAF mutation and N° of metastatic sites were prespecified as included variables before the study was performed. MV model in overall population included BRAF status and n° of metastatic sites. Prognostic variables included in the multivariable model were retained statistically significant if *p*-value ≤ 0.05 (**). The incidence and severity of irAEs, including gastrointestinal (9.5% vs. 20%, *p* = 0.34), endocrine (23.8% vs. 20%, *p* = 0.77), cutaneous (*p* = 23.8% vs. 10%, *p* = 0.24), and hepatic (23.8% vs. 30%, *p* = 0.65) toxicities, were similar between the two groups.

## Data Availability

All data relevant to the study are included in the article or uploaded as Supplementary Information.

## Supplementary Materials

The following supporting information can be downloaded at https://www.mdpi.com/article/10.3390/jcm15010069/s1. Methods S1: Inclusion and exclusion criteria and variable definitions [52]; Methods S2: Outcomes; Methods S3: Statistical analysis; Results Table S1: Sensitivity analysis with univariate and multivariate Cox proportional hazard models including all the variables; Table S2: Pre-specified Univariate PFS cox analysis on BRAF-mutants and BRAF WT population for AM vs PM administration; Table S3: Post-hoc Analysis: Sensitivity univariate PFS Cox analysis excluding patient “with brain metastasis” and “with more than 3 metastatic sites” for AM/PM subgroups; Table S4: Multivariate analysis of PFS and OS for AM/PM subgroups with individual variables and interaction tests; Table S5: Median Survival Levels Depending on Age; Table S6: Demographic characteristics according to patient’s age; Figure S1: PFS and OS according to patient age and time of administration.
