# Supplementary material for: ChronoimmunoTOX: A Single-Institution Retrospective Study on How the Time of Administration Impacts Immune Checkpoint Inhibitor Efficacy and Toxicity in Melanoma
Source: J Clin Med. 2025 Dec 22;15(1):69. doi: 10.3390/jcm15010069 (PMC12787149; doi:10.3390/jcm15010069)
Supplement: Supplementary file 1 [file jcm-15-00069-s001.zip › jcm-4016305-supplementary.pdf]

Supplementary Material

Methods

Methods S1: Inclusion and exclusion criteria and variable definitions …………..**2**

Methods S2: Outcomes……………………………………………………………… **3**

Methods S3: Statistical analysis……………………………… ………………………..**4**

Results

Table S1: Sensitivity analysis with univariate and multivariate Cox proportional hazard models including all the variables **5-6**

Table S2: Pre-specified Univariate PFS cox analysis on BRAF-mutants and BRAF WT population for AM vs PM administration……………………………………………..**7**

Table S3: Post-hoc Analysis: Sensitivity univariate PFS Cox analysis excluding patient "with brain metastasis" and "with more than 3 metastatic sites" for AM/PM subgroups…………………………..8

Table S4: Multivariate analysis of PFS and OS for AM/PM subgroups with individual variables and interaction tests **9**

Table S5: Median Survival Levels Depending on Age…….. …**10-11**

Table S6: Demographic characteristics according to patient's age…... …**12**

Figure S1: PFS and OS according to patient age and time of administration ……..**13**

Methods S1. Inclusion and exclusion criteria and variable definition

Inclusion criteria:

- Patients with histologically confirmed stage III or stage IV cutaneous or mucosal or acral or not otherwise specified (NOS) melanomadiagnosed between January 1st 2018 and September 1st 2024, treated with the combination of nivolumab and ipilimumab, either with the standard approved dose for metastatic melanoma patients (ipilimumab 3 mg/kg, nivolumab 1 mg/kg), or with the neoadjuvant dose, derived from PRADO trial [52] (ipilimumab 1 mg/kg, nivolumab 3 mg/kg)
- Patients with stage III disease were considered potentially resectable but declined primary surgery due to the extent of the procedure itself.
- patients who signed the general informed consent at the time of their treatment or diagnosis and in the case of deceased patients, we considered only who had no documented objection to the use of their data. Specifically, for deceased patients we applied the Article 34 of the Swiss Federal Act on Human Research (HRA), which allows the use of previously collected data for research purposes.

Exclusion criteria:

- Patients whose data were not evaluable or who explicitly objected to the use of their medical records.
- Uveal melanoma

Variable definitions

Treatment's best response was assessed via CT scan three months after treatment initiation. At our center, all initial radiological evaluations are routinely discussed by a multidisciplinary board, including a dedicated oncologic radiologist, to determine treatment response.

We categorized an infusion as "morning" (AM) if completely administered before 2:00 PM, considering a 1-hour delay from prescription to infusion. Patients who received at least two morning infusions during their first four doses of first-line treatment were classified into the AM group. Conversely, patients with fewer than two morning infusions were categorized into the "afternoon" (PM) group. Patients who received less than four infusions due to toxicity or progression were classified in the AM group if at least 50% of the doses received were in the "morning". Patients who received a single infusion were classified according to that single infusion. According to the data reported in the full text concerning pharmacodynamic and pharmacokinetic considerations, we restricted the classification of patients into AM and PM groups based solely on the timing of the first four doses of nivolumab and ipilimumab, excluding the time of administration of the nivolumab maintenance therapy, if performed.

All grades of IRAEs were included in the analysis.

Methods S2. Outcomes

Primary endpoint

- PFS

The primary endpoint was to assess weather ICI administration timing (AM versus PM group) could impact on PFS. PFS was defined as time from to the start of the treatment until disease progression to identification of radiographic progression, symptoms, initiation of a new treatment, or death, whichever occurred first or last follow up.

Secondary endpoints:

- Best response at first radiological evaluation
- OS
- Rates, types and grade of IRAEs, according to CTCAE v.5
- Use and management of systemic immunesoppressive therapy for IRAEs

We studied if ICI administration timing could impact on best response at first radiological evaluation, overall survival (OS), rates of immune-related adverse events (irAEs), types and grades of toxicity according to CTCAE v.5, and the requirement for systemic immunosuppressive therapy to manage toxicities. G2 irAEs were also classified together with G3 and G4 events, because they required the use of corticosteroids, in order to reduce misclassification bias.

Best response was evaluated according to RECIST criteria if available and by revision of multidisciplinary tumor board evaluation and categorized as "partial response (PR)," "complete response (CR)," "stable disease (SD)," or "disease progression (PD)." Objective Response Rate (ORR) was defined as the sum of PR and CR, while Disease Control Rate (DCR) included PR, CR, and SD. OS was defined as the time from start of therapy until death of any cause. Patients without a documented event were censored at their last follow-up visit.

Methods S3. Statistical analysis

Descriptive statistics, including mean and standard deviation for continuous variables and frequency and percentage for categorical variables, were used to summarize participant characteristics. Levene's test for homogeneity of variance was conducted to ensure equality of variances between the groups, while the normality of continuous variables was evaluated using the Shapiro-Wilk test. Parametric or non-parametric tests were selected accordingly. Pearson's chi-square test was employed to examine the association between categorical variables (patient characteristics, tumor features, time of ICI administration (AM versus PM group), best response per RECIST Criteria, and treatment-related toxicities) and the Kruskal–Wallis rank sum test for continuous measures. PFS and OS were estimated with the Kaplan–Meier method, and group comparisons were made using the log-rank test. All statistical comparisons were made with two-tailed tests.

Univariate analyses were performed using Cox proportional hazards models, incorporating "time of ICI administration," clinical and demographic relevant variables as independent variables. Subsequently, variables clinically expected to significantly affect PFS and OS outcomes were identified "a priori" (BRAF status and the number of metastatic sites) and included in the multivariate Cox model along with any variable found to be significant ($p \leq 0.05$) in the univariate analysis. Variables assessed in the univariate Cox analysis included age at diagnosis, disease extension (more or less than 3 sites of metastasis), sex at birth, BRAF mutation status, presence of brain metastases.

Multivariable Cox regression analysis for PFS and OS and test of interaction were performed together to evaluate the independent prognostic value of ICI administration timing and other variables on primary and secondary endpoints. We conducted sensitivity analysis by creating a multivariate Cox regression model for PFS and OS with "time of ICI administration" variable and each of the individual clinical and demographical relevant variables studied.

Exploratory analyses were conducted to assess whether "time of ICI administration" impact on PFS could differ between patients' subgroups.

The results are presented as hazard ratios (HR) with 95% confidence intervals (CI 95%). All statistical analyses were carried out using R statistical software version 4.4.1 and Jamovi statistical software version 2.5.6.

Table S1: Sensitivity analysis with univariate and multivariate Cox proportional hazard models including all the variables

S1 Table		PFS		OS	
**Prognostic variable**	**Levels**	**Univariate analysis**	**Multivariate analysis**	**Univariate analysis**	**Multivariate analysis**
**N°. 41**		**HR (95% CI), P-value**	**HR (95% CI), P-value**	**HR (95% CI), P-value**	**HR (95% CI), P-value**
**ECOG PS**	0-1	-	-		
	2	1.81 (0.53- 6.14) p= 0.34	3.69 (0.75-18.16) p= 0.341	0.87 (0.11-6.77), p=0.896	2.26 (0.19- 26.47) p= 0.517
**Age**	<60	-	-		
	≥60	1.33 (0.57-3.10) p = 0.51	2.39 (0.70-8.19) p=0.167	1.63 (0.54-4.94), p=0.389	1.44 (0.31-6.70), p=0.639
**Sex**	F	-	-		
	M	0.56 (0.24-1.30) p= 0.18	0.88 (0.35- 2.17) p= 0.776	0.73 (0.25-2.13), p=0.568	0.92 (0.27-3.05), p=0.885
**N° of Metastatic Sites**	≤3	-	-		
	>3	2.55 (1.10-5.96) p= 0.030	1.63 (0.52-5.04) p= 0.40	2.17 (0.72-6.54), p=0.168	0.72 (0.17-3.04), p=0.651
**BRAF V600 mutational status**	no	-	-		
	yes	1.75 (0.73 4.17) p= 0.21	0.94 (0.33-2.72) p= 0.912	1.35 (0.45-4.07), p=0.590	0.57 (0.14-2.38), p=0.439
**Brain Metastasis**	no				
	yes	1.58 (0.67-3.70) p=0.28	0.41 (0.11-1.49) p= 0.47	3.29 (0.98-11.03), p=0.053	1.58 (0.32-7.70), p=0.572
**Time of Administration**	PM				
	AM	0.29 (0.12-0.70) p=0.006**	0.15 (0.04-0.53) p=0.003*	0.25 (0.08-0.80), p=0.019*	0.07 (0.01-0.48), p=0.006*
**Test for interaction**	**P-Value**			**P-Value**	
**ECOG PS**	0.91853773			0.9982442	
**Age**	0.03507417			0.6042047	
**Sex**	0.36886599			0.4860618	
**N° of Metastatic Sites**	0.88450736			0.5091154	
**BRAF V600 Mutational status**	0.20251985			0.3014212	
**Brain metastasis**	0.19972882			0.7722933	
Abbreviations: OS= overall survival (from treatment start to					

death); PFS = progression free survival (from treatment start to first progression) CI = confidence interval; HR = hazard ratio; ECOG PS = Eastern cooperative oncology group performance status<br>Prognostic variables were included in the multivariable model if P-value ≤0.05*.<br>Prognostic variables included in the multivariable model were retained statistically significant if P-value ≤0.05 (**).	

Table S2: Pre-specified Univariate PFS cox analysis on BRAF-mutants and BRAF WT population for AM vs PM administration

**Univariate PFS Cox Analysis for BRAF-mut population**				
**Variable**	**Levels**	**n**	**HR, 95% CI, p value**	
**ICI timing**	PM	10 (55.6%)	….	
	AM	8 (44.4%)	0.20 (0.04-0.98, p=0.047)	
**Univariate PFS Cox Analysis for BRAF-wt population**				
**Variable**	**Levels**	**n**	**HR, 95% CI, p value**	
**ICI timing**	PM	3 (18.8%)	….	
	AM	13 (81.2%)	0.62 (0.06-5.97, p=0.677)	

Table S3: Sensitivity univariate PFS Cox analysis excluding patient “with brain metastasis” and “with more than 3 metastatic sites” for AM/PM subgroups

Cox Table excluding patient with brain metastasis

Explanatory	Levels	all	HR (Univariable)
AM/PM	PM	9 (40.9)	-
	AM	13 (59.1)	0.18 (0.05-0.68, p=0.011)

Cox Table- excluding patient with more than 3 metastatic sites

Explanatory	Levels	all	HR (Univariable)
AM/PM	PM	11 (39.3)	-
	AM	17 (60.7)	0.36 (0.11-1.14, p=0.083)

Table S4: Multivariate analysis of PFS and OS for AM/PM subgroups with individual variables and interaction tests

**S4 Table**		**PFS**	**OS**
**Prognostic variable**	**Levels**	**Multivariate analysis**	**Multivariate analysis**
**Total N. 41**		**HR (95% CI), P-value**	**HR (95% CI), P-value**
**Time of Administration *ECOG PS**	PM	-	-
	AM	0.29 (0.12-0.71, p=0.006	0.25 (0.08-0.80, p=0.019
**Time of administration *Age**	PM	-	-
	AM	0.30 (0.12-0.72, p=0.007	0.26 (0.08-0,83, p=0.024
**Time of administration *Sex**	PM		
	AM	0.31 (0.13-0.77, p=0.011	0.25 (0.08-0.81, p=0.021
**Time of administration *N. of Metastatic sites**	PM	-	-
	AM	0.35 (0.14-0.89, p=0.028	0.27 (0.08-0,95, p=0.041
**Time of Administration *BRAF V600 status**	PM	-	-
	AM	0.31 (0.12-0.79, p=0.014	0.20 (0.05-0,75, p=0.017
**Time of Administration *Brain metastasis**	AM	-	-
	PM	0.30 (0.12-0.70, p=0.006	0.30 (0.09-1.02, p=0.054
**Interaction test with time of Administration**	**P-Value**		**P-Value**
**ECOG PS**	0.91853773		0.9982442
**AGE**	0.03507417		0.6042047
**SEX**	0.36886599		0.4860618
**N° of Metastatic Sites**	0.88450736		0.5091154
**BRAF V600 Mutational status**	0.20251985		0.3014212
**Brain metastasis**	0.19972882		0.7722933
Abbreviations: OS= overall survival (from treatment start to death); PFS = progression free survival (from treatment start to first progression) CI = confidence interval; HR = hazard ratio; ECOG PS = Eastern cooperative oncology group performance status			

Tables S5: Univariate Cox analysis, Pairwise comparisons depending on Age and time of administration on PFS.

**5a. Univariable Cox Regression Analysis of PFS According to Age and Timing of administration**			
**Explanatory**	**Levels**	**all**	**HR (95%IC, Univariable)**
Age, Time of Administration	<60, AM	11 (26.8)	-
	<60, PM	11 (26.8)	12.05 (2.35-61.65, p=0.003)
	>60, PM	9 (22.0)	6.64 (1.25-35.16, p=0.026)
	>60, AM	10 (24.4)	5.17 (0.96-27.73, p=0.055)

**5b. Pairwise Comparisons Between Age/Timing Subgroups for PFS (Holm-Adjusted p-values)**		
**Levels**	**Levels**	**p-value**
<60, PM	<60, AM	0.003
<60, PM	<60, AM	0.115
>60, PM	<60, PM	0.655
>60, AM	< 60, AM	0.294
>60, AM	<60, PM	0.294
>60, AM	>60, PM	0.854
Note. p-value adjustement Method: holm		

**5c. Age-Stratified Univariable Cox Models for timing of immunotherapy administration.**			
**Explanatory**	**Levels**	**all**	**HR (Univariable)**
**< 60, AM vs. PM**	<60, PM	11 (50.0)	-
	<60, AM	11 (50.0)	0.09 (0.02-0.46, p=0.004)
**> 60, AM vs. PM**	>60, PM	9 (47.4)	-
	>60, AM	10 (52.6)	0.89 (0.25-3.17, p=0.854)

**5d. Survival analysis strafied on patients age**		
		**95% Confidence Interval**

	**Median (months)**	**Lower (months)**	**Upper (months)**
**Age < 60y**	21	10,27	NaN
Age ≥ 60y	23,2	3,3	NaN

Table S6: Demographic characteristics according to patient's age

S6 Table				
	**N**	**< 60 yo (N=22, %)**	**≥ 60 yo (N=19, %)**	**Test Statistic**
**Sex,** *M*	41	12 (50)	13 (70)	P=0.36[1]
**Number of Comorbidities**	41			
more than 1		0 (0)	6 (30)	P<0.01[1]
0		12 (50)	3 (20)	
1		10 (50)	10 (50)	
**Type of Melanoma**	40			
Cutaneous		18 (80)	12 (70)	P=0.14[1]
MUP		4 (20)	2 (10)	
Mucosal		0 (0)	1 (10)	
NOS		0 (0)	3 (20)	
**BRAF status,** *mutated*	41	16 (70)	7 (40)	P=0.02[1]
**Brain metastasis,** *presence*	41	6 (30)	13 (70)	P=0.01[1]
**More than 3 sites of metastasis**	41	7 (30)	6 (30)	P=0.99[1]
**ECOG PS**	41			
0		19 (90)	18 (90)	P=0.15[1]
1		3 (10)	0 (0)	
2		0 (0)	1 (10)	
**LDH at baseline**	41	217.9 297.0 364.0	199.7 268.0 343.0	P=0.84[1]
**S100 at baseline**	39	0.0 0.1 0.2	0.0 0.1 0.2	P=0.43[1]
**Time of administration,** *AM group*	41	11 (50)	10 (50)	P=0.87[1]
**N is the number of non-missing value. [1]Pearson.**				

Figure S1: Kaplan–Meier curves for progression-free survival (PFS) stratified by age (<60 vs ≥60 years) and time of immunotherapy administration (AM vs PM).

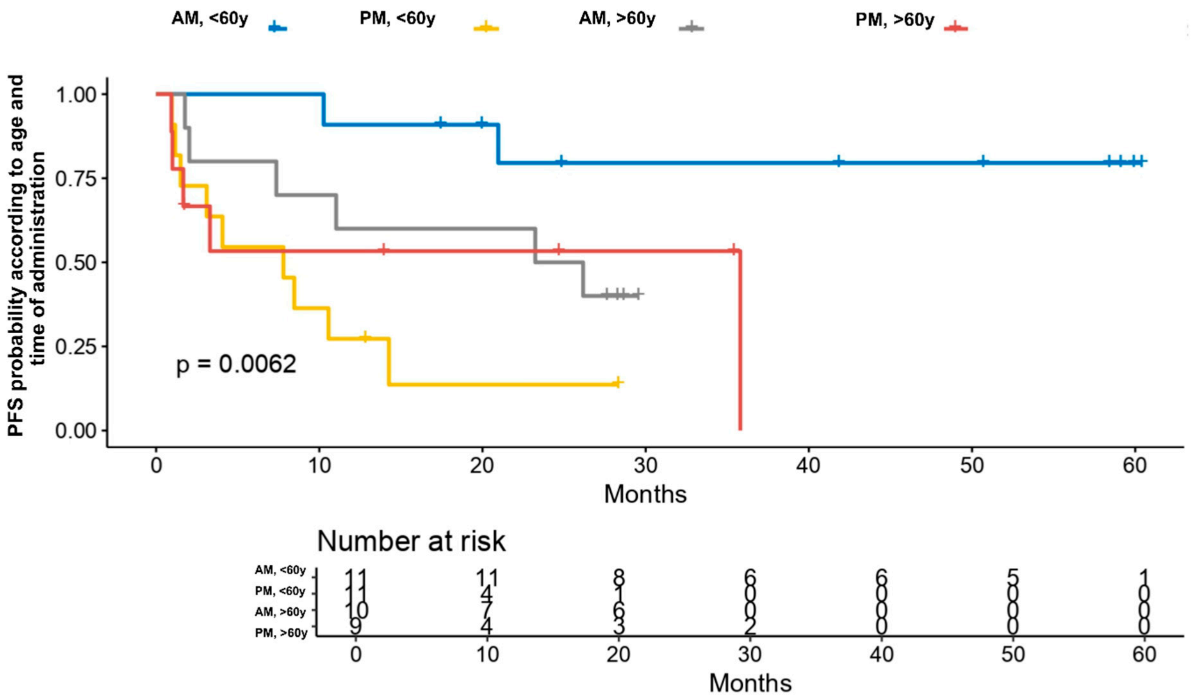

Figure S1. Patients younger than 60 years treated in the morning (AM, blue curve) showed the most favorable PFS outcomes. The log-rank test revealed a statistically significant overall difference among the four groups (**p = 0.0062**). The number at risk at each time point is shown below the graph. Subgroup analyses confirmed a significantly reduced risk of progression or death for patients <60 years treated in the morning compared to those treated in the afternoon (HR = 0.09, 95% CI: 0.02–0.46, $p = 0.004$). No significant timing effect was observed in patients aged ≥60 years
